# Indications and outcomes for intravitreal injection of C_3_F_8_ gas for symptomatic vitreomacular traction

**DOI:** 10.1038/s41598-021-97639-z

**Published:** 2021-09-10

**Authors:** Josef Guber, Celine Rusch, Ivo Guber, Hendrik P. N. Scholl, Christophe Valmaggia

**Affiliations:** 1 Eye Clinic, Cantonal Hospital Sankt Gallen, Rorschacherstrasse 95, 9007 Sankt Gallen, Switzerland; 2 Department of Ophthalmology, University of Geneva, Geneva, Switzerland; 3 Department of Ophthalmology, University of Basel, Basel, Switzerland; 4 Institute of Molecular and Clinical Ophthalmology Basel (IOB), Basel, Switzerland

**Keywords:** Retinal diseases, Risk factors

## Abstract

To evaluate the indications and outcomes of perfluoropropane (C3F8) gas injection for symptomatic vitreomacular traction (VMT). A retrospective analysis of eyes with VMT treated with 0.3 mL of C3F8 gas was performed. Patients were not asked to posture after gas injection. In phakic patients, cataract surgery was performed simultaneously. Patients were examined after one week and one month postoperatively. Twenty-nine consecutive eyes of 26 patients with symptomatic VMT who underwent pneumatic vitreolysis were included. A complete posterior vitreous detachment was achieved in 18 eyes (62.1%) after a single gas injection at the final visit. The rate of posterior vitreous detachment was reduced significantly with the presence of epiretinal membrane (ERM) (*p* = 0.003). Three eyes formed a macular hole (MH) postoperatively and another eye developed a retinal detachment. Mean visual acuity increased significantly after one month (p < 0.008). Pneumatic vitreolysis is a viable option for treating VMT with few adverse events. Patient with concomitant ERM had a significantly lower success rate.

## Introduction

The posterior vitreous detachment (PVD) is caused by normal age-related degeneration of the vitreous gel where the vitreous gel detaches from the posterior pole^1^.

Vitreomacular adhesion (VMA) has no effect on the retina, is usually asymptomatic and can be a transient phenom during physiologic PVD. However, persistent VMA can result in vitreomacular traction (VMT). A VMT can have impact on the macula causing retinal distortion, macular edema and macular hole (FTMH). Patients may complain about photopsia and distorted or reduced vision^2–4^.

Management options for VMT include observation, pars plana vitrectomy (PPV), enzymatic vitreolysis with ocriplasmin injection and new pneumatic vitreolysis (PVL).

PPV is up to now the gold standard in treatment of symptomatic VMT with high success rates and good postoperative visual acuity. Nonetheless, PPV is associated with some intra- and postoperative complications including cataract formation in phakic eyes, retinal detachment, endophthalmitis, and FTMH formation. Furthermore, pars plana vitrectomy is cost-intensive and often associated with general anaesthesia and hospitalisation^5–9^.

Pharmacologic induced PVD with intravitreal injection of ocriplasmin and integrin antagonist, has variable success rate in achieving VMT release of around 30–40%. The disadvantages of this approach are the relatively high costs and the frequent side effects like transient visual acuity loss and visual field constrictions with reduced electroretinogram responses, constricted retinal vessels, loss of outer retinal signals on OCT scans and pupillary anomalies^10–14^.

Intravitreal injection of gas, so called pneumatic vitreolysis (PVL) is a minimally invasive procedure to induce PVD and release VMT. This procedure is very cost-effective and has a low rate of postoperative complications^15–21^. Unpublished data showed significant higher release rates for C3F8 (84%) when com-pared with SF6 (56%) or air (14%). [Steinle N. Pneumatic vitreolysis for VMT: A comparison of intravitreal injections of C3F8 versus SF6 versus air. Paper presented at: American Society of Retina Specialists Annual Meeting; August 11–15, 2017; Boston, MA].

However, studies reviewing the indications and risk factors for failure and for com-plications are missing.

This retrospective study evaluates the indications, in particular the risk factors for failure, and outcomes after injection of C3F8 gas for symptomatic vitreomacular traction.

## Patients and methods

The study was approved by the Ethikkommission Ostschweiz (EKOS Nr. 19.015) and was performed as part of departmental quality control. All data were anonymized, therefore patient consent was waived by the Ethikkommission Ostschweiz (EKOS Nr. 19.015). The study adhered to the tenets of the Declaration of Helsinki.

**Figure 1:**
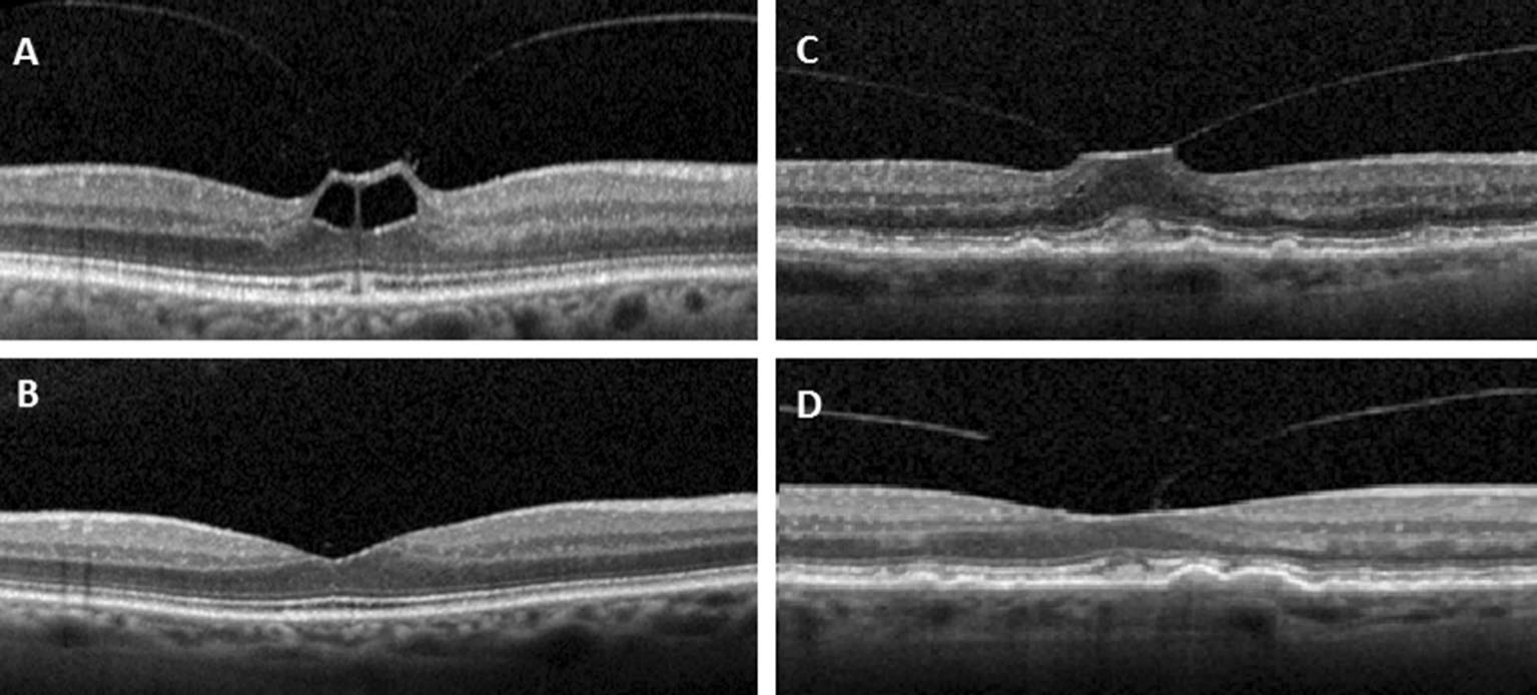
VMT preoperative and one month after C3F8 injection. From top to bottom: complete VMT release (**A**, **B**), partial VMT release with persistent VMA (**C**, **D**). *VMT* vitreomacular traction, *VMA* vitreomacular adhesion.

In this retrospective study, all patients who underwent a single C3F8 gas injection for symptomatic vitreomacular traction between January 2017 and June 2020 at the Eye Clinic of Sankt Gallen Cantonal Hospital (KSSG) were included. Exclusion criteria were history of vitrectomy or injections, history of laser photocoagulation to the macula, high myopia, aphakia, history of retinal detachment, retinal breaks or vitreous hemorrhage and obvious lattice areas. Each patient received a complete ophthalmological examination including fundus examination, best corrected distance visual acuity using Snellen charts (BCVA, in decimal) and spectral domain optical coherence tomography (SD-OCT, Spectralis HRA OCT, Heidelberg Engineering, Heidelberg, Germany) before surgery, at 1 week and 4 weeks after treatment. In general, every patient had a follow-up after 1 week and a second follow-up after 1 month. In case that the VMT release did not occur additional follow-ups usually took place. Maximal central retinal thickness (CRT) and maximal VMT adhesion diameter prior treatment was digitally measured using SD-OCT scans. Postoperative outcome was graded in complete VMT release, partial VMT release or no release at all using SD-OCT images. Partial release was defined as persistent VMA without traction (Fig. 1).

Data collection included patients’ demographics (gender, age, and laterality), best-corrected visual acuity (BCVA; in decimal), maximal central retinal thickness as well as maximal VMT adhesion diameters. Primary outcome was the release rate of VMT at 1 and 4 weeks and the final visit. Secondary outcomes were the change in BCVA and postoperative complications. After the cataract operation, the gas injection was carried out.

### Surgical technique

The patient is placed in rear supine position. The procedure is performed in topical anesthesia only using lidocaine eyedrops. The eye and the eyelids are cleaned with Betadine and a drape is used to cover the face. An eyelid speculum holds the eyelids open. Anterior chamber paracentesis with a MVR blade is performed to reduce intraocular pressure by controlled drainage of aqueous humor from the anterior chamber. The location of injection is marked using a caliper: 3–3.5 mm from the limbus. The injection of 0.3 ml of C3F8 gas is performed with a short 30-gauge needle tip usually in the supero-temporal quadrant. At the end of surgery topical antibiotic is administered into the eye. Postoperatively, the patient is asked not to lie flat on the back for 5 days. In phakic patients phacoemulsification was performed first followed by intravitreal gas injection. The procedures were addressed simultaneously by the same surgeon.

### Statistical analysis

Data was summarized with descriptive statistics. For numeric variables, medians and ranges are reported, and for categorical variables, number and percentages of patients in each category are reported. For outcome categories, Wilson 95% confidence intervals for the percentages are also given.

Associations between outcome categories and other factors were analyzed with Fisher's exact tests, those with numeric variables were analyzed using Kruskal–Wallis tests (three outcome categories) or Wilcoxon rank sum tests (two outcome categories).

### Ethics approval and consent to participate

The study was approved by the Ethikkommission Ostschweiz (EKOS Nr. 19.015) and was performed as part of departmental quality control. All data were anonymized, therefore patient consent was waived by the Ethikkommission Ostschweiz (EKOS Nr. 19.015). The study adhered to the tenets of the Declaration of Helsinki.

## Results

Twenty-nine eyes of 26 consecutive patients who underwent pneumatic vitreolysis with C3F8 gas were included. The age ranged between 60 and 92 years with a median age of 77.9 years. In 15 of 29 gas injections, the procedure was combined with a phacoemulsification. Eight eyes (27.6%) had concurrent epiretinal membrane and two eyes (6.7%) had a FTMH stage 2. Three eyes (10.3%) suffered from age-related macular degeneration (AMD) and one each eye (3.4%) suffered from mild non-proliferative diabetic retinopathy (NPDRP) and branch retinal vein occlusion (BRVO) with peripheral sectorial retinal laser, respectively. Patients’ characteristics are summarized in Table 1. The median duration of follow-up was 75 days (range 7–510 days). In one patient, the VMT release already occurred within 1 week and the visual acuity went from 0.8 preoperative to 1.0 within that week, so no additional follow-up took place.

**Table 1:** Patients’ characteristics.

Frequency of categories	N = 29	%
**Sex**		
Male	12	41.4
Female	17	58.6
**Eye**		
Right	10	34.5
Left	19	65.5
**Phaco**		
Yes	15	51.7
No	14	48.3

Numeric variables	Median	Range
Age (years)	77.9	60.8–92.7
BCVA (decimal)	0.4	0.05–0.8
CRT (μ)	361	234–790
VMT basis (μ)	454	152–1964

*BCVA* best-corrected visual acuity, *CRT* central retinal thickness, *VMT basis* vitreomacular traction adhesion diameter.

**Figure 2:**
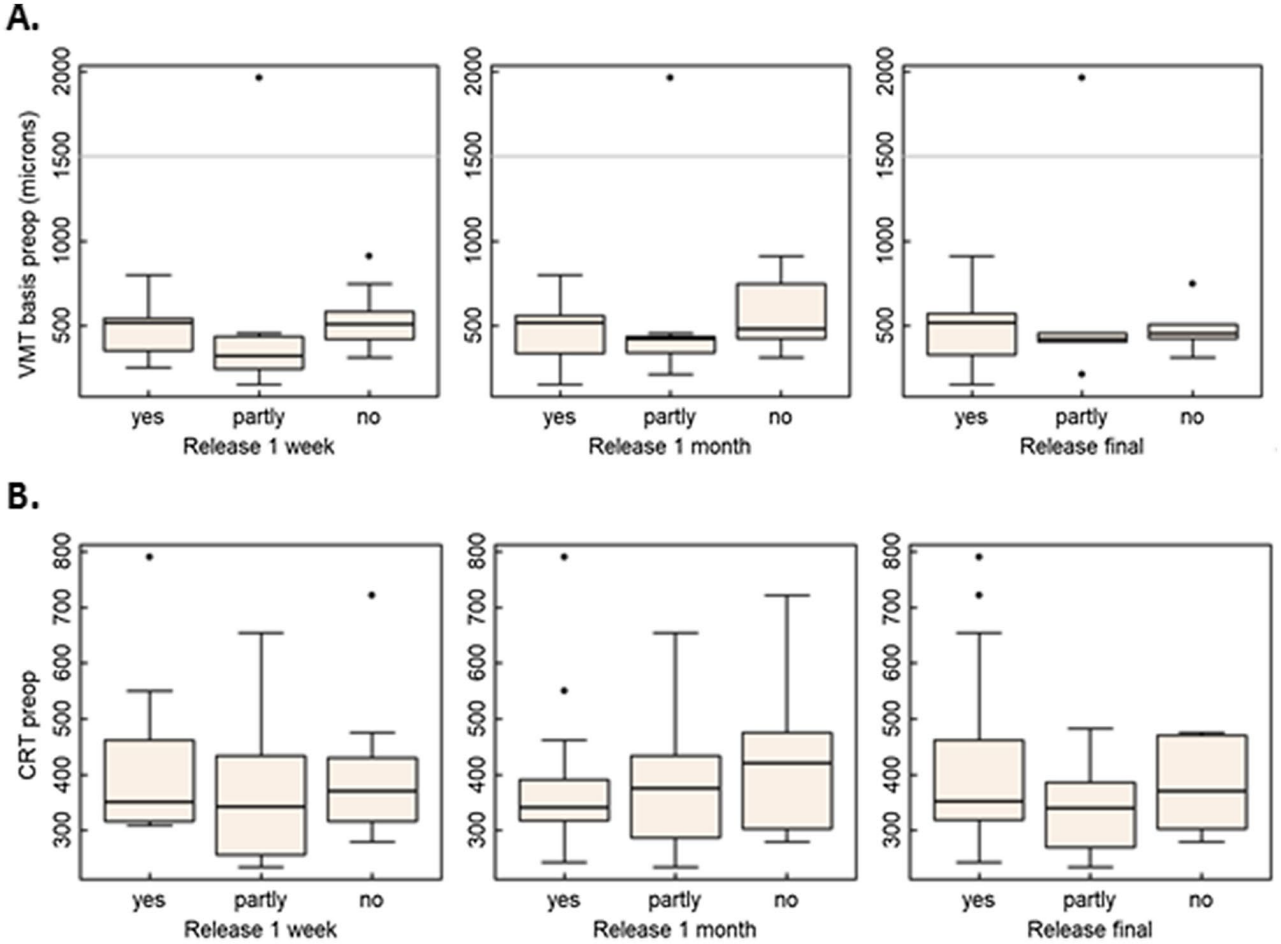
Association of VMT release with VMT Basis and CRT. Boxplots showing the lack of association between VMT release and VMT basis (µm). Grey lines indicate the threshold of 1500 µm. All VMT basis values except for one were clearly below this threshold (**A**). Boxplots showing the lack of association between CRT (µm) and VMT release (**B**).

At the final visit, a complete or partial VMT release was achieved in 24 out of the 29 eyes (82.8%). Only in five eyes (17.2%), no release at all was achieved. The exact rates of VMT release within the different follow-up times are displayed in Table 2. Subgroup analysis of VMT release in relation to patient’s characteristics and OCT findings did not show any correlation with age, gender, simultaneous phacoemulsification and maximal central retinal thickness or maximal VMT adhesion diameter, respectively. However, analysis of secondary diagnosis showed that patients with an accompanying ERM had a significant lower release rate (Table 3). The VMT basis threshold of 1500 microns, above which the risk of VMT release failure may be increased, had no impact on the VMT release rate (Fig. 2). Both patients with stage 2 macular holes experienced a successful VMT release with closure of the macular holes. In addition, the two patients with peripheral sectorial laser after BRVO and NPDRP achieved a complete VMT release.

**Table 2:** Rates of VMT release.

	1 week	1 month	Final visit
Complete release	10 (34.5%)	16 (55.2%)	18 (62.1%)
	19.9–52.7%	37.6–71.6%	44.0–77.3%
Partial release	8 (27.6%)	7 (24.1%)	6 (20.7%)
	14.7–45.7%	12.2–42.2%	9.8–38.4%
No release	11 (37.9%)	6 (20.7%)	5 (17.2%)
	22.7–56.0%	9.8–38.4%	7.6–34.5%

Number and (%) of patients with each outcome category, and 95% confidential interval (CI) for the percentage.

**Table 3:** VMT release in relation to patients’ characteristics, secondary diagnosis and OCT findings at each time point.

	VMT release	1 week	1 month	Final visit
Male = 12 Female = 17	Complete or partial release	8 (66.7%)	11 (91.7%)	11 (91.7%)
		10 (58.8%)	12 (70.6%)	13 (76.5%)
	No release	4 (33.3%)	1 (8.3%)	1 (8.3%)
		7 (41.2%)	5 (29.4%)	4 (23.5%)
Fisher's exact test	*p*-value	0.717	0.354	0.37
	odds ratio	0.71	0.22	0.30
Age (years) Median (range)	Complete or partial release	77.0 (60.8–86.2)	77.2 (60.8–86.6)	77.6 (60.8–86.6)
	No release	81.4 (72.4–92.7)	80.4 (77.9–92.7)	81.7 (77.9–92.7)
Wilcoxon test	*p*-value	0.12	0.11	0.10
Phaco Yes = 15 No = 14	Complete or partial release	9 (60.0%)	13 (86.7%)	13 (86.7%)
		9 (64.3%)	10 (71.4%)	11 (78.6%)
	No release	6 (40.0%)	2 (13.3%)	2 (13.3%)
		5 (35.7%)	4 (28.6%)	3 (21.4%)
Fisher's exact test	*p*-value	1	0.39	0.65
	Odds ratio	0.83	2.60	1.77
ERM No = 21 Yes = 8	Complete or partial release	15 (71.4%)	20 (95.2%)	20 (95.2%)
		3 (37.5%)	3 (37.5%)	4 (50.0%)
	No release	6 (28.6%)	1 (4.8%)	1 (4.8%)
		5 (62.5%)	5 (62.5%)	4 (50.0%)
Fisher's exact test	*p*-value	0.197	0.003	0.013
	Odds ratio^1^	3.9	27.3	17.2
VMT basis (μ) Median (range)	Complete or partial release	411 (152–1964)	420 (152–1964)	438 (152–1964)
	No release	507 (311–911)	480 (311–911)	454 (311–748)
Wilcoxon test	*p*-value	0.122	0.384	0.845
CRT (μ) Median (range)	Release (full or partial)	350 (234–790)	344 (234–790)	352 (234–790)
	No release	370 (280–721)	420 (280–721)	370 (280–475)
Wilcoxon test	*p*-value	0.808	0.581	0.933

*ERM* epiretinal membrane, *CRT* central retinal thickness, *VMT basis* vitreomacular traction adhesion diameter.

Mean visual acuity (mean preoperative BCVA = 0.39, decimal scale) increased significantly after one month (+ 0.13, *p*-value = 0.008) and at final visit (+ 0.20, *p*-value < 0.001).

**Figure 3:**
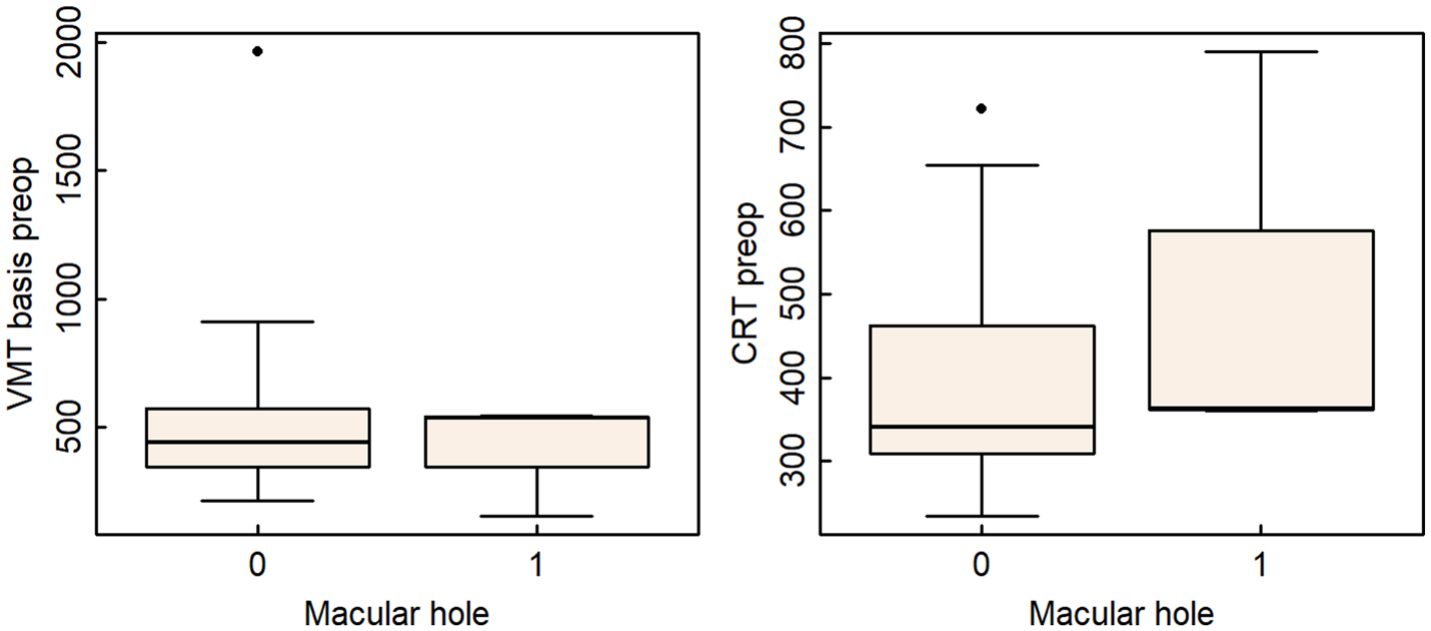
Complications in relation to VMT basis and CRT. Medians (ranges) of VMT basis (µm) and CRT (µm) for the three patients who developed a macular hole and the 26 others. There were no significant differences.

The visual acuity improved at least one line in 23 eyes (79.3%) and remained stable in 5 eyes (17.2%) at the final visit. In one eye (3.4%) visual acuity worsened from 0.4 to 0.1 after developing a macula-involving retinal detachment. In total, adverse events occurred in 4 out of 29 eyes (13.7%). Additionally to the retinal detachment, three eyes (10.3%) developed a macular hole after successful VMT release. However, this was not associated with increased central retinal thickness (CRT) or VMT adhesion diameter (Fig. 3).

## Discussion

Pneumatic vitreolysis (PVL) by intravitreal gas injection was introduced by Chan et al.^15^. The gas bubble should liquefy the vitreous body during the gas expansion and induce the cortical vitreous to collapse during the absorption phase of the bubble, leading to PVD. Recent studies have shown PVD rates ranging from 40 to 88.6% depending on the intravitreal gas administered^16–21^. In the present study, we analyzed the outcomes after a single 0.3 ml C3F8 gas injection as a treatment for VMT. A complete release could be achieved in 16 out of 29 (55.2%) within 1 month and in 18 out of 29 (60.9%; 95% CI 40.8–77.8) at the final visit. In another 6 eyes (20.7%) a partial release occurred until the final visit. Overall, in 24 out of 29 eyes (82.8%) some sort of VMT release could be achieved. Only in five eyes (17.2%) the injection did not lead to any VMT release. The mean time until VMT resolution is consistent with the majority of previous studies that also reported a VMT release within the first month after treatment in most cases^16–19^.

The mean ages of previous studies included in the meta-analysis by Yu et al. was between 51.4 and 76.6 years whereas our study had a mean age of 77.9 years^20^. This could be a possible reason for our moderate success rate, since Chan et al. reported significantly higher release rates in younger patients. In fact, they even reported age to be the strongest predictor for success when compared to better baseline visual acuity, absence of diabetes mellitus and female gender^19^. However, we could not show any significant association between age and success rate in our study. Furthermore, according to Chan et al. female gender is a negative predictor for PVL success (2017)^19^. We were also not able to show a significant association between gender and success rate in our study.

OCT findings are often used as inclusion or exclusion criteria whether certain treatments should be performed or not. We investigated the prediction value of the VMT adhesion diameter and central retinal thickness for the success rate for VMT release but no significant association between these prediction values and the VMT release rate could be found. However, previous studies suggested that large and diffuse VMT diameter could lead to a lower success rate and are therefore risk factors for failure for VMT resolution^16,18,19^.

In our study, patients with epiretinal membrane had significant lower VMT release rates at one month and at the final visit. Chan et al. showed also a trend of association of success rate and lack of epiretinal membrane, however, the results did not reach statistical significance^19^. In contrast to our findings, Yu et al. were not able to show a significant association between lower success rate and the presence of epiretinal membrane^20^. However, we noticed that patients with VMT and ERM still suffered from metamorphopsia postoperatively despite successful VMT resolution.

The two patients with a stage 2 macular hole and VMT were successfully treated with PVL leading to a complete VMT release with macular hole closure. The successful closure of stage 2 macular holes after PVL is consistent with multiple previous studies^15–19^. Some studies suggest that stage 2 macular holes and a lack of diabetes mellitus are predictors of success in VMT release^18,19^.

For other secondary ocular diagnoses such as BRVO, diabetic retinopathy and AMD, no significant difference in the VMT release rate could be detected, likely due to the small number of patients included in this study.

We also analyzed whether or not simultaneous cataract operation had a positive impact on release rates, but surprisingly no significant association was found.

Since all of these studies including ours are of retrospective nature, confounding factors may have led to wrong conclusions about the prediction value of these measurements for the success rate for VMT release.

Adverse events associated with PVL occurred in four eyes (13.7%) in the present study. Macular holes developed in three eyes (10.3%) and retinal detachment occurred in one eye (3.4%). Pars plana vitrectomy was successfully performed in two patients with macular and one patient with retinal detachment. One patient with macular hole denied further treatment because of systemic risk factors.

Similar rates adverse events have been reported in various previous studies^18–20^. Most common adverse event is a macular hole formation (10.3%) postoperatively. However, this is comparable with the natural history where 12% of all patients with VMT will develop a macular hole^22^. In addition, postoperative macular hole formation are reported in 9.8% after PPV and in 8.6% % after ocriplasmin injection, respectively^8,11^.

No systemic complications occurred in our study, which is in line with previous studies who have not reported any systemic complications either^15–21^.

In conclusion our study suggests that PVL is a viable option for treatment of symptomatic VMT with reasonable resolution and similar complication rates when compared with natural history, ocriplasmin injection or vitrectomy. However, VMT associated with epiretinal membrane has a significant lower success rate and pars plana vitrectomy with ERM peel might be considered as the first line option. However, further larger and prospective studies are needed to support our findings.

## Data Availability

The datasets used and/or analysed during the current study are available from the corresponding author on reasonable request.
